# USP30 promotes the progression of breast cancer by stabilising Snail

**DOI:** 10.1038/s41417-023-00718-8

**Published:** 2023-12-25

**Authors:** Kai Sun, Shichong Liao, Xinrui Yao, Feng Yao

**Affiliations:** 1 Department of Breast and Thyroid Surgery, Renmin Hospital of Wuhan University, Wuhan, Hubei P. R. China; 2 School of Science, University of Sydney, Sydney, Australia

**Keywords:** Breast cancer, Cell biology

## Abstract

Breast cancer (BC) is the most prevalent tumour in women worldwide. USP30 is a deubiquitinase that has been previously reported to promote tumour progression and lipid synthesis in hepatocellular carcinoma. However, the role of USP30 in breast cancer remains unclear. Therefore, we investigated its biological action and corresponding mechanisms in vitro and in vivo. In our study, we found that USP30 was highly expressed in breast cancer samples and correlated with a poor patient prognosis. Knockdown of USP30 significantly suppressed the proliferation, invasion and migration abilities of BC cells in vitro and tumour growth in vivo, whereas overexpression of USP30 exhibited the opposite effect. Mechanistically, we verified that USP30 interacts with and stabilises Snail to promote its protein expression through deubiquitination by K48-linked polyubiquitin chains and then accelerates the EMT program. More importantly, USP30 reduced the chemosensitivity of BC cells to paclitaxel (PTX). Collectively, these data demonstrate that USP30 promotes the BC cell EMT program by stabilising Snail and attenuating chemosensitivity to PTX and may be a potential therapeutic target in BC.

## Introduction

Breast cancer is the most common tumour in women worldwide and has a high mortality rate [1, 2]. Since 2004, the incidence of breast cancer has been increasing yearly, but the mortality rate has been gradually decreasing [3]. Although this indicates that some progress has been made in the treatment of breast cancer, the mortality rate of breast cancer remains high due to tumour progression and resistance to treatment [4]. Therefore, the development of new biomarkers and therapeutic strategies is essential for the precision treatment of breast cancer.

Epithelial-mesenchymal transition (EMT) is a process by which epithelial cells acquire mesenchymal cell functions and characteristics. In cancer, EMT can promote tumorigenesis, invasion, and metastasis and counteract tumour therapy [5]. EMT-associated transcription factors may redefine the epithelial state of cells and may also allow differentiated cancer cells to reacquire cancer stem cell properties, which condition the spread of solid tumours and the migration of tumour cells after basement membrane rupture [6]. Cancer cells develop plate-like and invasive footprints and express matrix metalloproteinases that degrade extracellular matrix proteins to enhance motility and invasiveness. Moreover, EMT may promote early tumour escape and thus promote drug resistance during tumour treatment [7].

Snail is a key transcription factor in EMT, Snail represses epithelial marker genes and activates genes associated with the mesenchymal phenotype. Snail can reduce the levels of E-cad and claudins and upregulate the levels of N-cad, MMP2, MMP9 and TWIST, which reshapes the structure of cancer cells and enables them to gain motility and invasive ability [2, 8, 9]. GSK-3β was first found to bind to Snail and reduce Snail degradation, thus regulating tumour progression [10]. In recent years, Snail has been shown to be highly expressed in breast cancer as an early marker of malignant phenotype and prognosis, and Snail is essential for cancer-associated fibroblast activation and promotion of tumour-initiating cell expansion in mouse breasts [11–13]. And increasing number of molecules have been shown to regulate breast cancer proliferation, invasion and migration through the regulation of Snail [14–16]. Ubiquitin-mediated degradation of Snail has also been reported to regulate the process of EMT [2].

USP30 is a deubiquitinating enzyme that was first identified in the nervous system. USP30 deubiquitinates TOM20, thereby limiting PINK1/PARKIN-mediated mitochondrial autophagy and restricting BAX/BAK-induced apoptosis, and is considered a potential target in Parkinson’s disease [17, 18]. In recent years, USP30 was also shown to be pro-carcinogenic in oral squamous cell carcinoma and associated with poor prognosis [19]. USP30 has been reported to promote lipid accumulation and tumour progression by stabilising ACLY in hepatocellular carcinoma [20, 21], and the recruitment of USP30 stabilises DRP1 to promote hepatocarcinogenesis [20]. However, knowledge of the physiological function of USP30 in breast cancer is limited.

In this study, we validated for the first time that USP30 plays a role in breast cancer, demonstrated that USP30 could combine with and deubiquitinates Snail and extensively investigated the role of USP30 in vivo and in vitro. In this study, we elucidated the expression of USP30 and its association with prognosis. In addition, we revealed the role of USP30 in breast cancer progression, EMT progression and deubiquitination of EMT marker proteins by studying and analysing cellular models, mouse models and human breast cancer samples. In addition, we explored the specific mechanisms of USP30 in breast cancer progression and EMT progression. Finally, we demonstrated the effect of USP30 on chemotherapy sensitivity. In summary, we investigated the role and mechanism of USP30 and Snail in breast cancer progression, deubiquitination-related processes and chemotherapy sensitivity.

## Methods

### Antibodies and reagents

Primary antibodies for Western blotting and immunohistochemical staining included anti-Snail (Santa Cruz sc-271977), anti-USP30 (Santa Cruz sc-515235), anti-β-actin (Cell Signaling Technology ab6276, clone AC-15), anti-FLAG (Roche MFCD02262912), and anti-ubiquitin (Santa Cruz Biotechnology sc-271289). The negative control siRNA (D-001810-01-05) and Snail siRNA (J-017386-06-0002) were purchased from GenePharma. USP30 siRNA was synthesised and purchased from GenePharma (forward: 5′-GCUGCUUGUUGGAUGUCUUTT-3′; reverse: 5′-AAGACAUCCAACAAGCAGCTT-3′).

### Cellular proliferation, invasion and migration assays

To measure cell proliferation, a Cell Counting Kit-8 (CCK-8) assay was performed according to the manufacturer’s instructions (CK04, Tongrento, Japan). Transwell assays were performed using Matrigel (normal DMEM:Matrigel = 9:1) or without Matrigel to measure cell invasion and migration. Equal amounts of cells were inoculated in serum-free medium in the upper chamber (Corning, USA), and DMEM containing 10% FBS was added to the lower chamber. Twenty-four hours later, the invaded cells were fixed and stained with 0.1% crystalline violet.

### Cell culture and stable cell lines

MDA-MB-231 and MCF7 cell lines(authenticated by STR profiling and tested for mycoplasma contamination) were cultured in DMEM (Gibco) supplemented with foetal bovine serum (10%; NEWZERUM), penicillin/streptomycin (1%; Corning), and amphotericin (0.2%; Corning). Plasmids or siRNAs were transfected using Lipofectamine 2000 (Invitrogen) following the manufacturer’s instructions. To generate stable cell lines, puromycin was used to select the infected cells.

### Coimmunoprecipitation assay

Two days after cotransfection of USP30-HA plasmids, MDA-MB-231 and MCF7 cells were lysed with 500 μL of IP lysis buffer (50 mM Tris-Cl at pH 7.4, 150 mM NaCl, 1 mM EDTA, 1% Triton X-100) with protease inhibitors (Roche 11836170001). Five percent of the input samples were saved and loaded for Western blotting. After incubation with mouse FLAG antibody (Santa Cruz Biotechnology sc-8036) overnight at 4 °C, 30 μL of prewashed protein A/G beads (Santa Cruz Biotechnology sc-2003) were added and incubated for 1 h. After four washes, the beads were pelleted and boiled with SDS loading buffer for Western blotting analysis.

### Quantitative real-time qPCR

The primer sequences used were as follows: SNAI1 (forward: 5′-CGAACTGGACACACATACAGTG-3′; reverse: 5′-CTGAGGATCTCTGGTTGTGGT-3′), USP30 (forward: 5′-GCUGCUUGUUGGAUGUCUUTT-3′; reverse: 5′-AAGACAUCCAACAAGCAGCTT-3′), and GAPDH (internal control, forward: 5′-TGCACCACCAACTGCTTAGC-3′; reverse: 5′-GGCATGGACTGTGGTCATGAG-3′).

### Ubiquitination assay

Two days after siRNA transfection or 3 days after infection with a control USP30-containing wild-type lentivirus, cells were treated with 20 µM MG132 for 6 h to block proteasomal degradation. Cells were then lysed with 150 µL denaturing lysis buffer (pH 6.8, 50 mM Tris-Cl at 1.5% SDS), and protein samples were collected by scraping and then boiled for 15 min. Ninety microlitres of denatured protein samples were added to 1.2 mL EBC/BSA buffer (pH 6.8, 180 mM NaCl, 0.5% NP40, 0.5% BSA) under 50 mM Tris-Cl), incubated overnight with anti-SNAI1 antibody or anti-FLAG antibody and incubated with protein A/G magnetic beads for 1 h at 4 °C. Then, polyubiquitinated SNAI1 was detected in the IP samples using a ubiquitin antibody.

### Cycloheximide pulse-chase assay

Cells were inoculated at 1 × 10^5^ to 2 × 10^5^ cells/well in 12-well plates and incubated overnight prior to the addition of cycloheximide (CHX). Cells were treated with 50 µg/mL CHX for 1-12 h as directed prior to protein blotting analysis.

### Animal studies

All animal experiments were performed in accordance with the Declaration of Helsinki and approved by the Animal Ethics Committee of Wuhan University. For the proliferation assay, MDA-MB-231 cells stably expressing control shRNA (shCtrl) or USP30 shRNA were generated. Four-week-old female BALB/c nude mice were randomly assigned to the following two groups: control group (injected with shCtrl cells) and knockdown group (injected with USP30 shRNA cells), each group includes 10 mice to ensure adequate power to detect a pre-specified effect. Cells (2.5 × 10^6^ cells per mouse) were inoculated subcutaneously into the right iliac fossa of the mice. Six weeks later, the mice were sacrificed, and the xenografts were removed for analysis.

### Immunohistochemistry staining and scoring

Eighty-eight paraffin-embedded samples of human breast cancer and 10 normal breast tissues were collected from Renmin Hospital of Wuhan University. The diagnosis was confirmed by histopathological examination, and detailed clinicopathological features are shown in the clinical records and pathology reports. The patients were followed up for a minimum of 5 years. All human participants in this study provided informed consent, and all methods were approved by the Institutional Ethics Committee of Renmin Hospital of Wuhan University. Immunohistochemistry (IHC) staining and evaluation were performed as described previously. The main steps of the procedure included dewaxing, antigen repair, closure, primary antibody incubation, washing, secondary antibody incubation, washing, DAB staining, washing, closure, and observation. The following antibodies were used for IHC analysis: anti-USP30 (Santa Cruz, sc-515235), anti-E-cadherin (Cell Signaling Technology, #14472), anti-N-cadherin (Cell Signaling Technology, #13116), and anti-Ki67 (Santa Cruz, sc-23900).

### Statistical analysis

Statistical analyses were performed using GraphPad 8.0 and SPSS 20.0 software. Correlations between USP30 and clinicopathological characteristics of breast cancer patients were calculated by Pearson’s χ2 test or Fisher’s exact test. Survival outcomes were assessed by the Kaplan‒Meier method and log-rank test. Correlations between USP30 and EMT markers in tissues were calculated by Pearson’s or Spearman’s correlation coefficients. Multiple group comparisons were performed by one-way ANOVA. These data represent at least three independent experiments and are expressed as the means ± SDs. P < 0.05 was considered to indicate statistical significance.

## Results

### USP30 is highly expressed in breast cancer and associated with patient prognosis

**Fig. 1:**
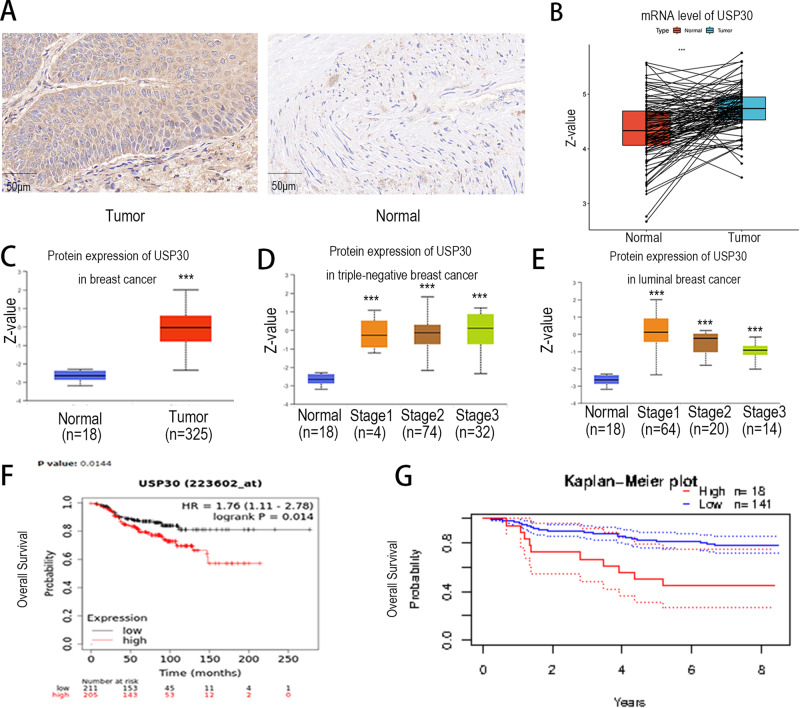
USP30 expression is higher in breast cancer than in normal tissue and higher USP30 predicts worse prognosis. **A** USP30 expression in breast cancer samples and normal breast tissues was detected by an immunohistochemistry (IHC) assay (800x). **B** USP30 mRNA levels are higher in breast cancer than in normal tissues. **C** USP30 protein levels are higher in breast cancer than in normal tissues. **D** Protein expression of USP30 at different stages of triple-negative breast cancer. **E** Protein expression of USP30 at different stages of luminal breast cancer. **F**, **G** High USP30 expression is correlated with poorer survival in breast cancer patients. (Figure 1B were compared by using Gene Ontology (GO) analyses and examined using the R “clusterProfiler” tool. Figure 1C–E were recovered from https://ualcan.path.uab.edu/; Fig. 1F were recovered from https://kmplot.com/analysis/; Fig. 1G were recovered from http://dna00.bio.kyutech.ac.jp/PrognoScan/index.html.). The values are the mean ± SD from three independent experiments. ns*P* > 0.05, *P < 0.05, **P < 0.01, ***P < 0.001 vs the corresponding group.

To verify the clinical relevance of USP30 to breast cancer, we examined USP30 expression in 100 breast cancer tissues and 10 normal breast tissues. Figure 1 shows that USP30 is highly expressed in breast cancer tissues; specifically, USP30 is overexpressed in tumours but rarely expressed in adjacent normal tissues (Fig. 1A). We compared USP30 mRNA expression levels between breast cancer and normal cohorts by using Gene Ontology (GO) analyses and examined using the R “clusterProfiler” tool, the results showed higher USP30 mRNA expression in the tumour group. Bioconductivity analysis showed that USP30 protein expression levels were high in breast cancers of different molecular stages (Fig. 1C–E). (Fig. 1C–E were recovered from https://ualcan.path.uab.edu/).

In addition, we analysed survival outcomes. The results showed that USP30-negative breast cancer patients had a longer disease-free survival time than USP30-positive patients (overall survival). (Fig. 1F were recovered from https://kmplot.com/analysis/; Fig. 1G were recovered from http://dna00.bio.kyutech.ac.jp/PrognoScan/index.html.) These results suggest that USP30 is highly expressed in breast cancer patients and is associated with poor patient prognosis.

### USP30 promotes breast cancer cell migration and invasion

**Fig. 2:**
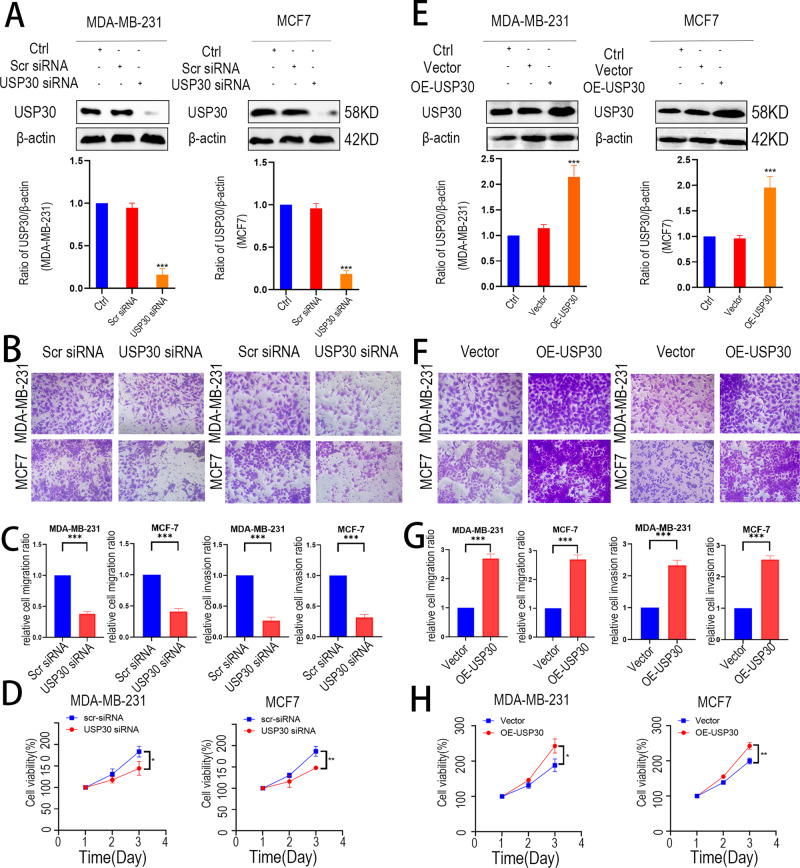
Knockdown of USP30 inhibits proliferation, invasion and migration of breast cancer cells, overexpression of USP30 has the opposite effect. **A** Knockdown efficiency of USP30 in MDA-MB-231 and MCF-7 cell lines. **B** Transwell assays showing that USP30 knockdown suppresses the invasion (left) and migration (right) of MDA-MB-231 and MCF-7 cells. **C** Relative invasion and migration ratio of MDA-MB-231 and MCF-7 cells in B. **D** CCK-8 assay showing that USP30 knockdown inhibits the proliferation of MDA-MB-231 and MCF-7 breast cancer cells. **E** Overexpression efficiency of USP30 in MDA-MB-231 and MCF-7 cell lines. **F** Transwell assays showing that USP30 overexpression induces the invasion and migration of MDA-MB-231 and MCF-7 cells. **G** Relative invasion and migration ratio of MDA-MB-231 and MCF-7 cells in **F**. **H** CCK-8 assay showing that USP30 overexpression promotes the proliferation of breast cancer cells (MDA-MB-231 and MCF-7 cells). The values are the mean ± SD from three independent experiments. ns*P* > 0.05, *P < 0.05, **P < 0.01, ***P < 0.001 vs the corresponding group.

To further demonstrate the role of USP30 in breast cancer, we overexpressed USP30 in MCF-7 and MDA-MB-231 cells using plasmids, and we knocked down USP30 using siRNA. In addition, CCK-8 assays and Transwell stromal gel assays were performed to confirm the function of USP30 in breast cancer. USP30 protein levels were similar between control and scrambled siRNA cells. USP30 expression was significantly lower in the USP30 siRNA group in both cell lines compared to the control and scrambled siRNA groups. The CCK-8 results indicated that USP30 knockdown inhibited cell proliferation. Silencing USP30 significantly reduced the relative cell invasion and migration rates compared with those of the control group (Fig. 2A–D).

In addition, we examined the effects of USP30 overexpression in MCF-7 and MDA-MB-231 cells to strengthen our conclusions. We transfected both cell lines with Flag-USP30 and Flag-NC plasmids as the overexpression and control groups. Cell proliferation was significantly increased in the overexpression group compared to the control group. Invasion and migration assays were also performed and showed that USP30 overexpression enhanced the prometastatic phenotype in breast cancer cell lines. In addition, salvage experiments showed that USP30 overexpression increased cell proliferation and the prometastatic phenotype in USP30 KD cells. These results suggest that USP30 promotes proliferative and prometastatic phenotypes, such as invasion and migration, in breast cancer cells (Fig. 2E–H). Our results suggest that USP30 promotes the proliferation, invasion and migration of breast cancer cells.

### USP30 regulates the progression of breast cancer by triggering EMT

**Fig. 3:**
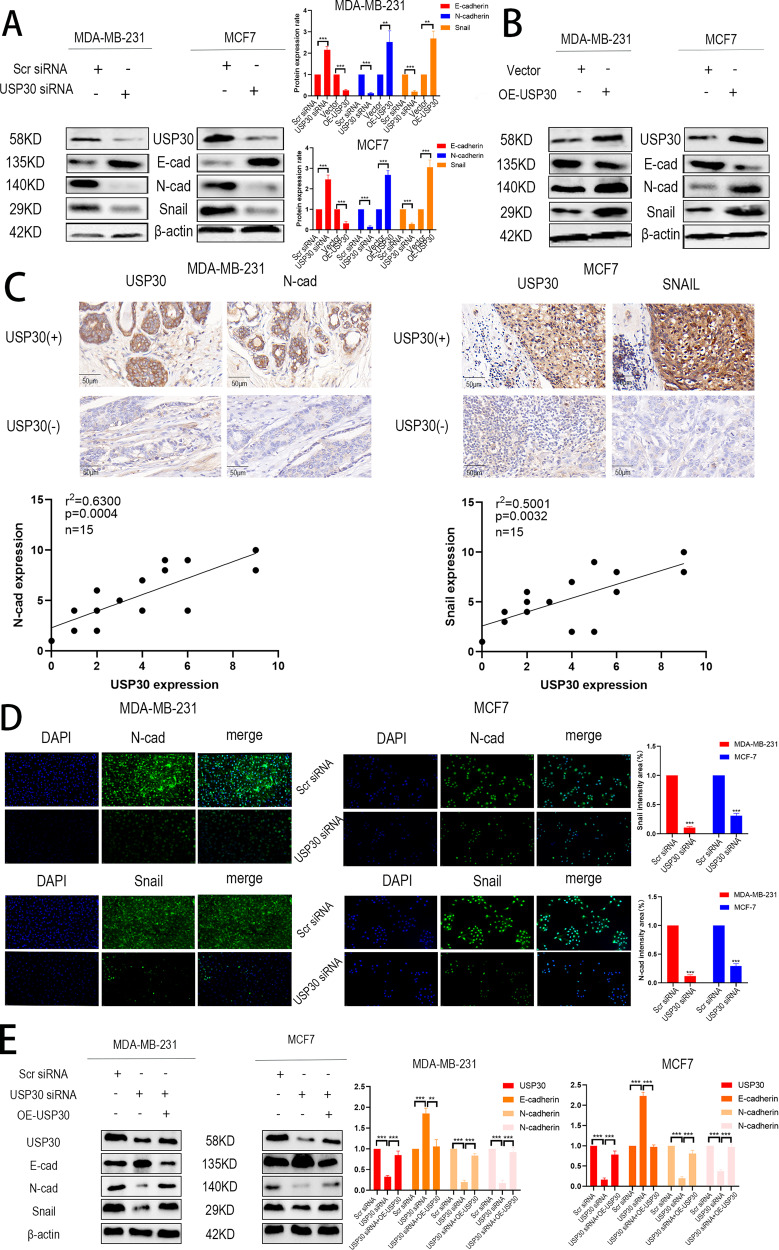
Knockdown of USP30 inhibits the expression of key indicators in EMT, overexpression of USP30 has the opposite effect. **A, B** Western blots of the EMT-related markers E-cadherin, N-cadherin, and Snail in USP30^KD^ and USP30^OE^ MDA-MB-231 and MCF-7 cells. **C** Representative IHC staining images of USP30 and EMT-related markers in breast cancer samples (400x). **D** Immunofluorescence images of N-cad and Snail staining in USP30^KD^ MDA-MB-231 and MCF-7 cells. **E** USP30 was overexpressed in USP30^KD^ cells, and the EMT-related markers E-cadherin, N-cadherin and Snail were detected by Western blot analysis. The values are the mean ± SD from three independent experiments. ns*P* > 0.05, *P < 0.05, **P < 0.01, ***P < 0.001 vs the corresponding group.

To determine whether USP30 contributes to EMT, we assessed EMT-associated protein expression by protein blotting, immunofluorescence and IHC staining. USP30 knockdown resulted in elevated expression of epithelial phenotypes, including E-cadherin, as well as decreased expression of N-cadherin and Snail in both cell lines. In contrast, overexpression of USP30 resulted in a mesenchymal phenotype characterised by decreased expression of E-cadherin and increased expression of N-cadherin and Snail compared to controls (Fig. 3A, B). Consistently, IHC staining of consecutive breast cancer sample sections supported these findings. In two consecutive sections of one sample, we observed that high expression of USP30 correlated with high expression of N-cadherin and Snail, whereas low expression of USP30 had the opposite result (Fig. 3C). Immunofluorescence results suggested that USP30 promotes the nuclear translocation of Snail and N-cad (Fig. 3D). Interestingly, we transfected USP30 knockdown cells with Flag-USP30 to rescue USP30 protein expression and assessed whether USP30 overexpression could reverse the changes in EMT. As shown in Fig. 3, the rescue experiments partially restored the expression of N-cadherin and Snail and reversed the increase in E-cadherin expression (Fig. 3E). Thus, our findings suggest that USP30 plays a crucial role in triggering EMT in breast cancer.

### USP30 knockdown inhibits tumour growth in vivo

**Fig. 4:**
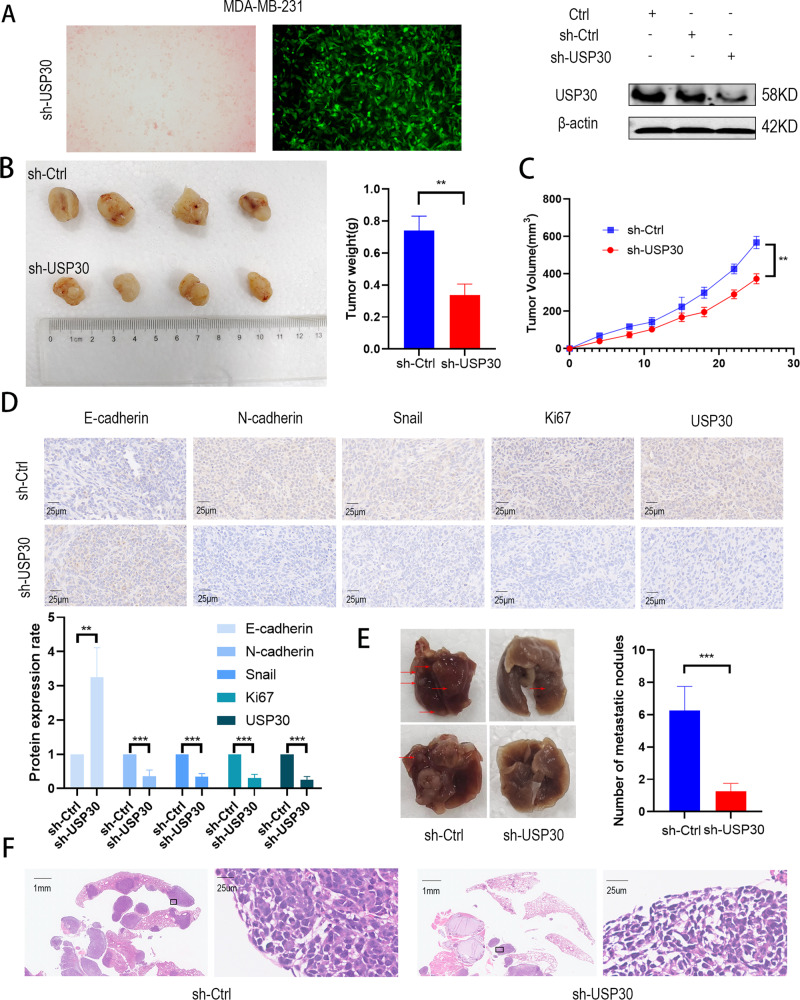
Knockdown of USP30 inhibits the tumor growth and lung metastasis of mice. **A** Efficiency of USP30 siRNA transfection for stable knockdown in MDA-MB-231 cells. **B** Comparison of tumour weights among various groups (*n* = 4). **C** Average tumour volume of the mice in the two groups. **D** Representative IHC staining of EMT-related markers and Ki67 in tumours of different groups. Knockdown of USP30 resulted in elevated E-cadherin and decreased N-cadherin, Snail and Ki67. **E** Comparison of lung metastasis among various groups (*n* = 4). **F** Representative HE staining of lung tissues of different groups. The values are the mean ± SD from three independent experiments. ns*P* > 0.05, *P < 0.05, **P < 0.01, ***P < 0.001 vs the corresponding group.

In view of the in vitro results, we then established an in vivo xenograft model to validate the role of USP30 in breast cancer growth and metastasis. First, MDA-MB-231 cells were infected with LV-shUSP30 or LV-shCtrl to assess the growth of tumours in both groups (Fig. 4A). After a period, we removed, photographed and weighed the transplanted tumours to assess the growth of the xenografts (Fig. 4B). The volume of tumours derived from shUSP30 cells was significantly lower than that of the corresponding control tumours (Fig. 4C). Representative IHC staining of the different groups is shown in Fig. 4D. We observed lung metastases in both groups of mice and found that the sh-USP30 group had significantly fewer lung metastases than the sh-Ctrl group (Fig. 4E, F). This finding validates that USP30 promotes breast cancer growth in vivo.

### USP30 interacts with and stabilises Snail through deubiquitination

**Fig. 5:**
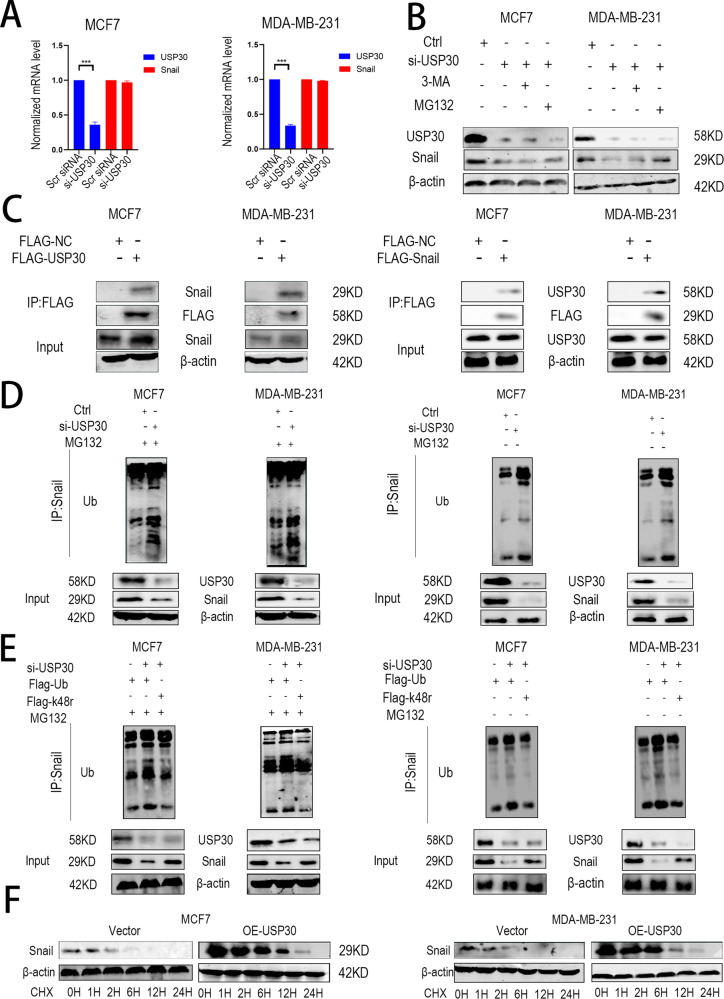
USP30 interacts with Snail and inhibits k48 ubiquitination degradation of Snail. **A** mRNA expression of USP30 and Snail in USP30^KD^ MDA-MB-231 and MCF-7 cells. **B** Autophagy inhibitors and proteasome inhibitors were applied, and these experiments demonstrated that knockdown of USP30 stabilises Snail by deubiquitination instead of by inhibiting autophagy. **C** Flag-USP30, Flag-Snail and Flag-NC were transiently transfected into MCF7 and MDA-MB-231 cells, and co-IP assays were performed. **D** Knockdown of USP30 leads to increased ubiquitination of Snail. **E** Flag-Ub and Flag-K48R were transfected into USP30^KD^ MDA-MB-231 and MCF-7 cells, and the ubiquitination levels were determined. **F** USP30 plasmids were transiently transfected into MCF7 and MDA-MB-231 cells for 48 h. The resulting cells were treated with 50 µg/ml CHX for the indicated times, and the cells were prepared for Western blot analysis to examine Snail protein levels. Greyscale analysis of Snail expression was conducted, and actin protein expression was used as an internal control. The values are the mean ± SD from three independent experiments. ns*P* > 0.05, *P < 0.05, **P < 0.01, ***P < 0.001 vs the corresponding group.

Because USP30 has a relatively large effect on Snail and Snail can be degraded by ubiquitin, the mechanism by which it regulates Snail is of interest. A PCR analysis showed that USP30 did not regulate Snail through transcription (Fig. 5A). We added MG132 and 3-MA to inhibit proteasomal degradation and autophagy of Snail, respectively, and found that MG132, but not 3-MA, could rescue the reduction of Snail caused by knockdown of USP30, suggesting that USP30 stabilises Snail through the proteasomal pathway but not the autophagic pathway (Fig. 5B). To evaluate the interaction between Snail and USP30, we performed a series of immunoprecipitation experiments. As shown in the figure, Snail was detected in USP30 immunoprecipitates but was absent in the FLAG control samples. Additionally, we performed reverse IP verification, USP30 was detected in Snail immunoprecipitates but was absent in the FLAG control samples, which demonstrated that USP30 and Snail are bound in breast cancer cells (Fig. 5C). We knocked down USP30 in both cell lines separately with or without MG132 treatment and observed the ubiquitination level of the substrate Snail. We found that the ubiquitination level was higher in the USP30^KD^ groups (Fig. 5D). We then examined the ubiquitination status of Snail in the presence of Flag-Ub and Flag-k48r in USP30^KD^ cells, and the results showed that USP30 inhibits k48 ubiquitination degradation of Snail (Fig. 5E). Chx assays showed that Snail was degraded gradually over the course of treatment (Fig. 5F). Taken together, these results reconfirm that the Snail-stabilising effect of USP30 is mediated through its DUB activity.

### Knockdown of Snail rescues the progression of breast cancer in USP30^OE^ cells

**Fig. 6:**
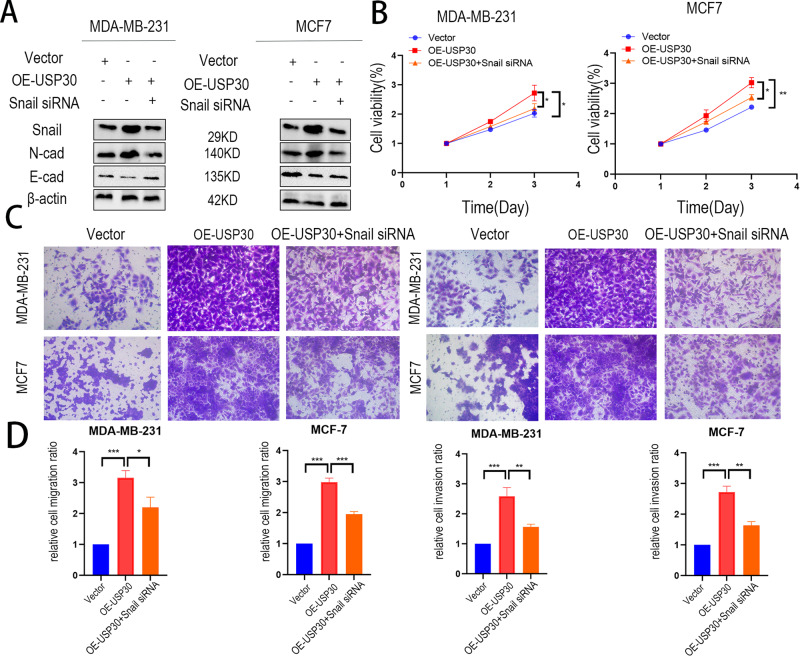
Knockdown of Snail inhibits progression of USP30 overexpression breast cancer cells. **A** Knockdown efficiency of Snail, N-cadherin and E-cadherin in USP30^OE^ MDA-MB-231 and MCF-7 cells. **B** CCK-8 assay showing that Snail knockdown inhibited breast cancer cell proliferation in USP30^OE^ MDA-MB-231 and MCF-7 cell lines. **C** Transwell assays showing that Snail knockdown inhibited breast cancer cell invasion in MDA-MB-231 and MCF-7 cells. **D** Relative invasion and migration ratio of MDA-MB-231 and MCF-7 cells. The values are the mean ± SD from three independent experiments. ns*P* > 0.05, *P < 0.05, **P < 0.01, ***P < 0.001 vs the corresponding group.

We then performed reverse experiments to verify that knockdown of Snail rescues the proliferation, invasion and migration of USP30^OE^ cells. Snail-siRNA was transfected into USP30^OE^ MCF7 and MDA-MB-231 cells, and the expression of Snail was assessed (Fig. 6A). CCK-8 assays and transwell assays with or without Matrigel were performed to assess the proliferation, invasion and migration of breast cancer cells (Fig. 6B–D).

### USP30 diminishes the sensitivity of breast cancer cells to PTX

**Fig. 7:**
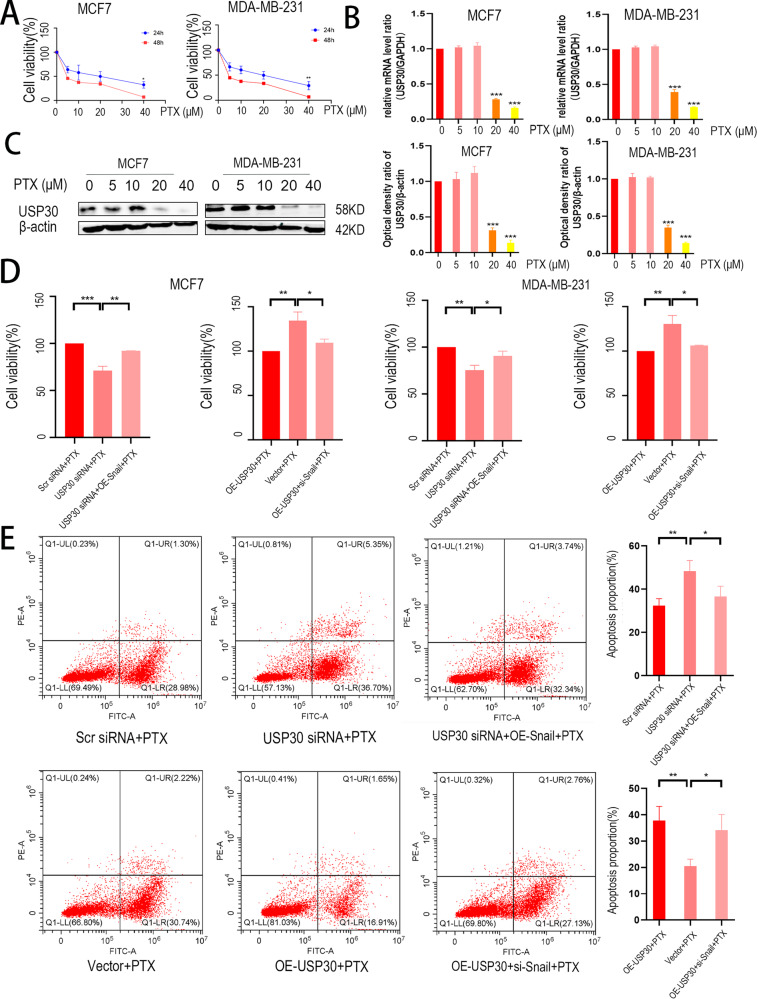
Knockdown of USP30 promotes PTX-induced apoptosis in breast cancer cells, overexpression of USP30 has the opposite effect. **A** The proliferation of two breast cancer cell lines after PTX treatment at different concentrations (0, 5, 10, 20 or 40 µM) and for 24 h was detected by CCK-8 assay. **B** The mRNA levels of USP30 in breast cancer cells treated with different concentrations of PTX (0, 5, 10, 20 or 40 µM) were measured by qRT‒PCR. **C** Protein blot analysis showing the protein expression of USP30 in breast cancer treated as described above. Bottom: Quantitative analysis of the optical density ratio of USP30 to β-actin. **D** Cell viability was assessed by CCK-8 assay after USP30 knockdown, overexpression and PTX treatment (20 µM). **E** Analysis of apoptosis with FACS in MDA-MB-231 cells treated as described above. The values are the mean ± SD from three independent experiments. ns*P* > 0.05, *P < 0.05, **P < 0.01, ***P < 0.001 vs the corresponding group.

We investigated the effect of USP30 on the sensitivity of breast cancer chemotherapy because USP30’s effect on breast cancer treatment. MCF-7 and MDA-MB-231 cells were suppressed by PTX in a concentration- and time-dependent manner (Fig. 7A). We treated MCF7 and MDA-MB-231 cells with different concentrations of PTX (0, 5, 10, 20 or 40 µM) for 24 h and observed that the mRNA and protein levels of USP30 increased or remained unchanged after treatment with the higher concentrations (0-10 µM) and decreased with the concentration of 20-40 µM (Fig. 7B, C). A CCK-8 assay showed great differences in cell viability between the USP30 knockout and control groups at 24 h, but in the Snail overexpression group, the inhibitory effect of PTX on breast cancer cell proliferation was significantly inversed (Fig. 7D). To further validate the role of USP30-Snail axis on PTX sensitivity, we assayed cell activity by flow cytometry (Fig. 7E). All our results suggest that PTX-induced apoptosis was partially mediated by USP30/Snail axis in breast cancer.

## Discussion

Breast cancer is the most common cause of cancer-related death in women, and there seems to have been no breakthrough in the treatment of breast cancer in recent years; thus, new pathways and targets are urgently needed [3, 4, 22]. Deubiquitinating enzymes have also been shown to be increasingly associated with cancer [23]. In our study, we identified the role of USP30 in breast cancer and explored its mechanism of action. Our study showed that USP30 is highly expressed in breast cancer and is associated with poor prognosis in breast cancer. In vitro experiments verified that knockdown of USP30 inhibited proliferation and EMT in breast cancer, and its overexpression yielded the opposite result. In vivo experiments showed that knockdown of USP30 inhibited tumour growth. Subsequently, our mechanistic experiments verified that USP30 regulates breast cancer progression and EMT by binding to Snail and deubiquitinating Snail. Moreover, our study showed that USP30 decreased the chemosensitivity of PTX in breast cancer.

There are more than 100 predicted deubiquitinases in the human genome. DUBs cleave ubiquitin chains from proteins, in turn regulating the function of these substrates [24, 25]. USP30 has been proven to act as a key inhibitor of mitophagy by counteracting the action of parkin, and USP30 belongs to the peptidase C19 family [26, 27]. USP30 has been proven to play a role as a cancer inducer in previous studies. USP30 is highly expressed in both hepatocellular carcinoma and oral squamous cell carcinoma and is associated with poor prognosis [19–21], but the role of USP30 in breast cancer was previously unclear. In our study, we found that USP30 promotes the progression of breast cancer, which is consistent with previous studies.

In the EMT process of tumours, E-cadherin is downregulated, while Snail and N-cadherin are upregulated, which can reduce cell adhesion, enhance invasion and migration, and lead to a poorer prognosis [28]. Upregulation of USP30 can facilitate this process to promote EMT and facilitate tumour invasion and migration. In our study, we found that knockdown of USP30 decreased the viability and suppressed the proliferation and migration of breast cancer cells; conversely, overexpression of USP30 increased the viability and promoted the proliferation and migration of breast cancer cells. Deletion of Snail eliminated these effects, which indicates that USP30 regulates the progression of breast cancer via EMT.

Snail plays a crucial role in EMT, and elevated levels of Snail can promote the growth, mobility, survival and invasion of cancer cells [29]. The SRD domain in Snail can be easily modified, such as by phosphorylation, glycosylation, acetylation and ubiquitination. Among these modifications, Snail is highly sensitive to ubiquitination and proteasomal degradation, and inhibition of Snail ubiquitination can stabilise Snail at the protein level [30–37]. Previous studies showed that USP26 maintains the stability of Snail by removing the K48-linked ubiquitin chain and thereby promotes oesophageal squamous cell carcinoma metastasis [30]. DUB3 promotes breast cancer metastasis by deubiquitinating Snail [35]; thus, we further explored the mechanism by which USP30 regulates Snail. In our study, we demonstrated the binding of USP30 and Snail in breast cancer cells for the first time and showed that USP30 inhibits k48 ubiquitination degradation of Snail and thus positively regulates Snail. Our study also demonstrated that USP30 diminishes the chemosensitivity of PTX in breast cancer, which suggests that USP30 may be a potential target in breast cancer.

The limitation of our study is that the specific loci of USP30 action with Snail were not investigated, and deeper mechanistic studies need to be added. We will continue to pay attention to this issue and perform further studies.

In conclusion, we identified Snail as a specific substrate of USP30 and showed that USP30 stabilises Snail via its deubiquitinase activity, which promotes breast cancer metastasis and chemosensitivity.

## Data Availability

The datasets generated during and/or analysed during the current study are available from the corresponding author on reasonable request.
